# An Extremely Rare Case of Synchronous Low-Grade Polymorphous Adenocarcinoma With Canalicular Adenoma of the Minor Salivary Gland of the Palate

**DOI:** 10.7759/cureus.36591

**Published:** 2023-03-23

**Authors:** Srinidhi Kasthurirengan, Rubin S John

**Affiliations:** 1 Oral and Maxillofacial Surgery, Saveetha Dental College and Hospital, Chennai, IND

**Keywords:** multiple salivary gland tumours, palate, minor salivary gland, canalicular adenoma, polymorphous adenocarcinoma

## Abstract

Tumors of the salivary gland are a group of complex, heterogeneous lesions that are located either in the parotid gland, submandibular gland, sublingual gland, or minor salivary glands. These tumors have a wide range of etiology, pathophysiology, treatment, and prognosis. Multiple salivary gland tumors are extremely rare and usually occur more commonly in major salivary glands than in minor glands. A 61-year-old man with a chief complaint of swelling in the upper jaw for the past eight years reported to the department of oral and maxillofacial surgery. Incisional biopsy revealed a canalicular adenoma (CA) of the minor salivary gland of the palate. Wide local excision was done with closure using a buccal pad of fat and a collagen sheet. Surprisingly, the excisional biopsy was suggestive of synchronous low-grade polymorphous adenocarcinoma (PAC) with CA of the minor salivary gland of the palate. This appears to be the first reported case of PAC with CA found in the palate.

## Introduction

Polymorphous adenocarcinoma (PAC) is the second most common intraoral malignant salivary gland tumor [1]. It occurs two times higher in women than men. The mean age of occurrence is 59 years. The most common site is the palate followed by buccal mucosa, retromolar region, upper lip, and base of the tongue. In rare instances, it can occur in major salivary glands or lacrimal glands. It is usually a painless swelling that may present with bleeding, ulceration, or telangiectasia. The overall survival rate is good [1,2].

A canalicular adenoma (CA) is a benign salivary gland tumor composed of ductal epithelial cells arranged in cords. The age of occurrence ranges from 40 to 70 years [2]. It occurs more commonly in men than women. The commonest site of occurrence of CA is the minor salivary glands, especially in the upper lip. In rare instances, it can occur in the buccal mucosa or palate. It is an asymptomatic painless lesion, which is usually an incidental finding [3]. Multiple salivary gland tumors can occur as synchronous or metachronous and can occur on one side or both sides. Occurrence of multiple salivary gland tumors is very rare and it occurs either as benign or malignant. Here, we present an unusual case of synchronous PAC, the second most common malignant neoplasm of minor salivary glands, which affects more commonly the palate and rarely the upper lip, associated with CA, a benign neoplasm exclusive of minor salivary glands, which frequently affects the upper lip.

## Case presentation

A 61-year-old male patient came to the department of oral and maxillofacial surgery with a chief complaint of swelling in the upper jaw for the past eight years. The patient had a history of diabetes for the past three years. The patient was a known smoker for 20 years but had quit the habit 10 years back. The patient did not have any other comorbidities. According to the patient, the swelling was small when it started and has attained its present size gradually over the course of eight years.

**Figure 1:**
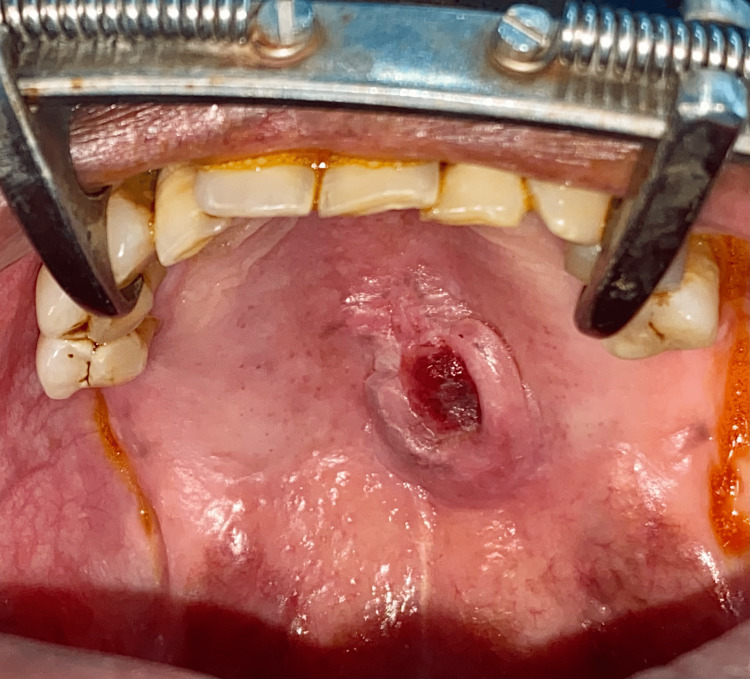
Preoperative image of the tumor The image shows a lesion measuring 2.1 x 0.9 x 0.6 cm in the mid-palatal region with the presence of ulceration in the center.

On examination, there was a lesion measuring 2.1 x 0.9 x 0.6 cm in the mid-palatal region with the presence of ulceration in the center (Figure 1). The patient did not have any pain or bleeding. The lesion was present from the level of the second premolars till the anterior part of the junction of the hard and soft palate.

**Figure 2:**
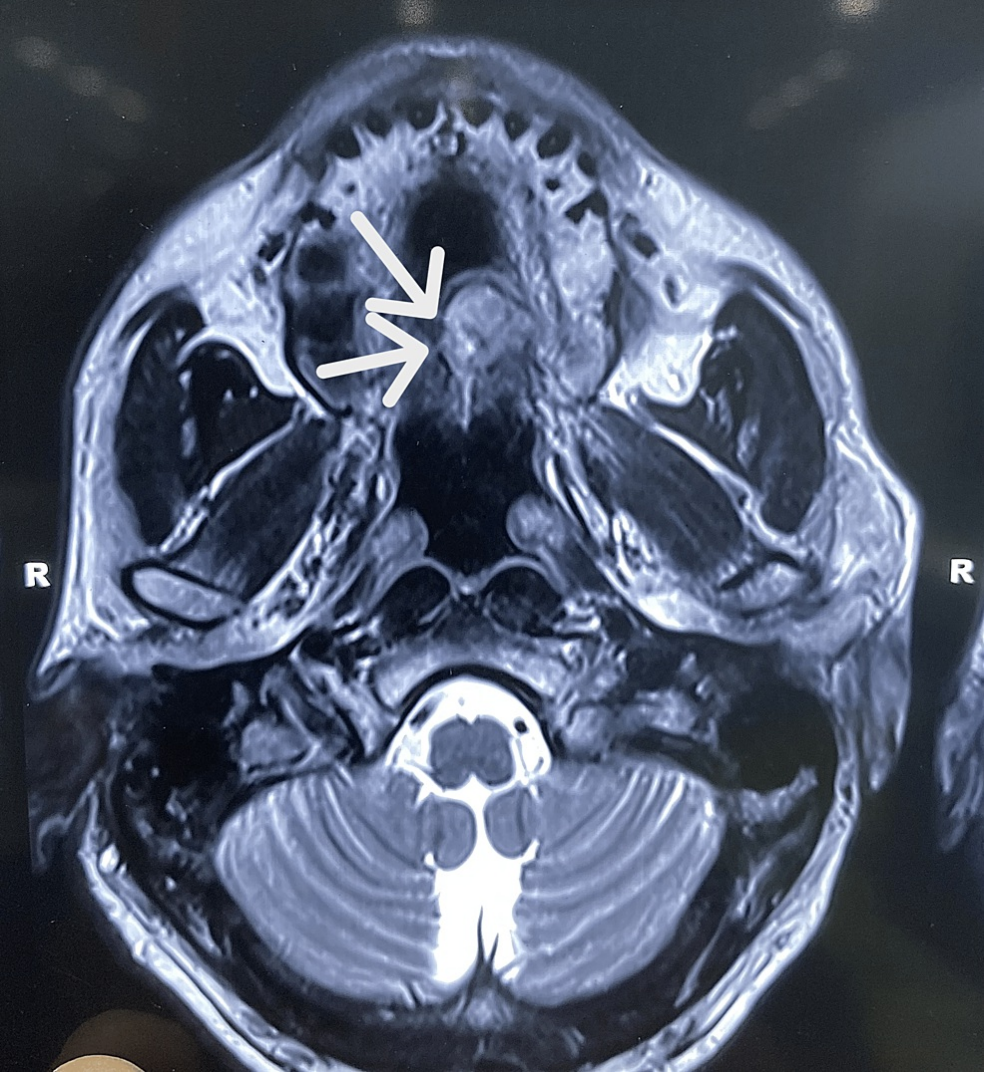
Radiograph showing the lesion in the palate The arrows in the contrast CT show a well-defined lesion on the left side of the palate.

**Figure 3:**
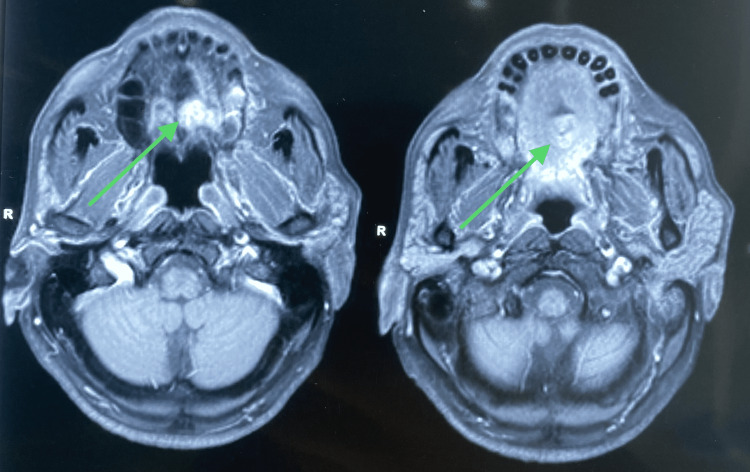
Radiograph showing axial sections of the lesion on the palate The arrows in the CT show a lesion on the palate.

**Figure 4:**
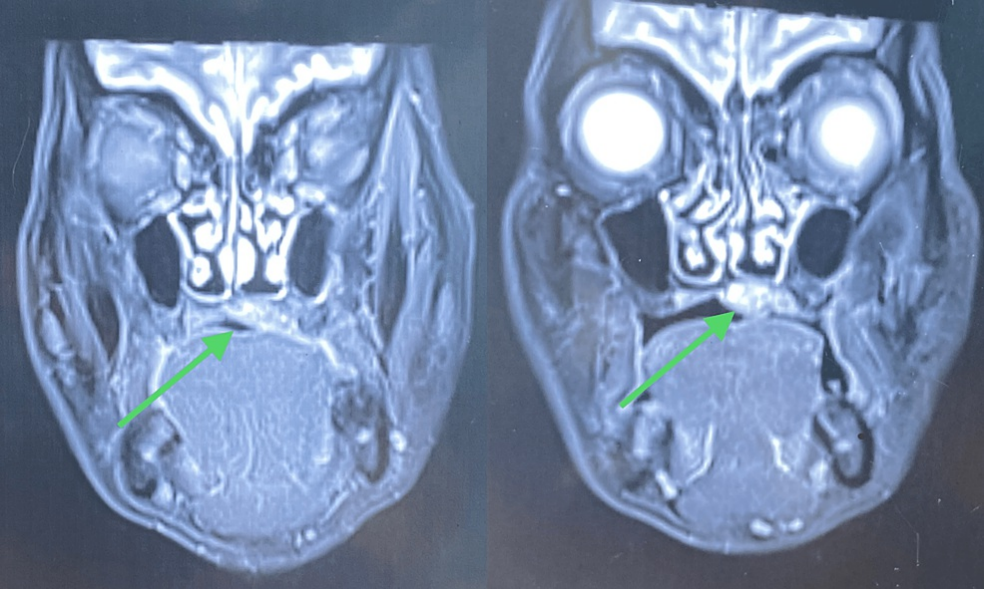
Radiograph showing coronal sections of the lesion on the palate The arrows in the CT show a lesion on the palate.

On radiographic examination, contrast CT revealed a radio-opaque lesion in the mid-palatal region, which did not involve the maxillary bone (Figures 2-4). The left greater palatine canal was involved.

An incisional biopsy was performed for the lesion, which was suggestive of CA. Sections showed neoplasm of glandular origin showing epithelial tumor cells arranged in anastomosing branching cords and tubules, which appear to float in the stroma. The tumor cells were cuboidal to columnar showing moderate amounts of amphophilic cytoplasm and monomorphous basophilic nuclei. The intervening stroma was loose and edematous with rich vascularity, moderate inflammatory infiltrate, and extensive hemorrhagic areas. Parakeratinized stratified squamous epithelium of variable thickness suggestive of the surface epithelium was also seen.

**Figure 5:**
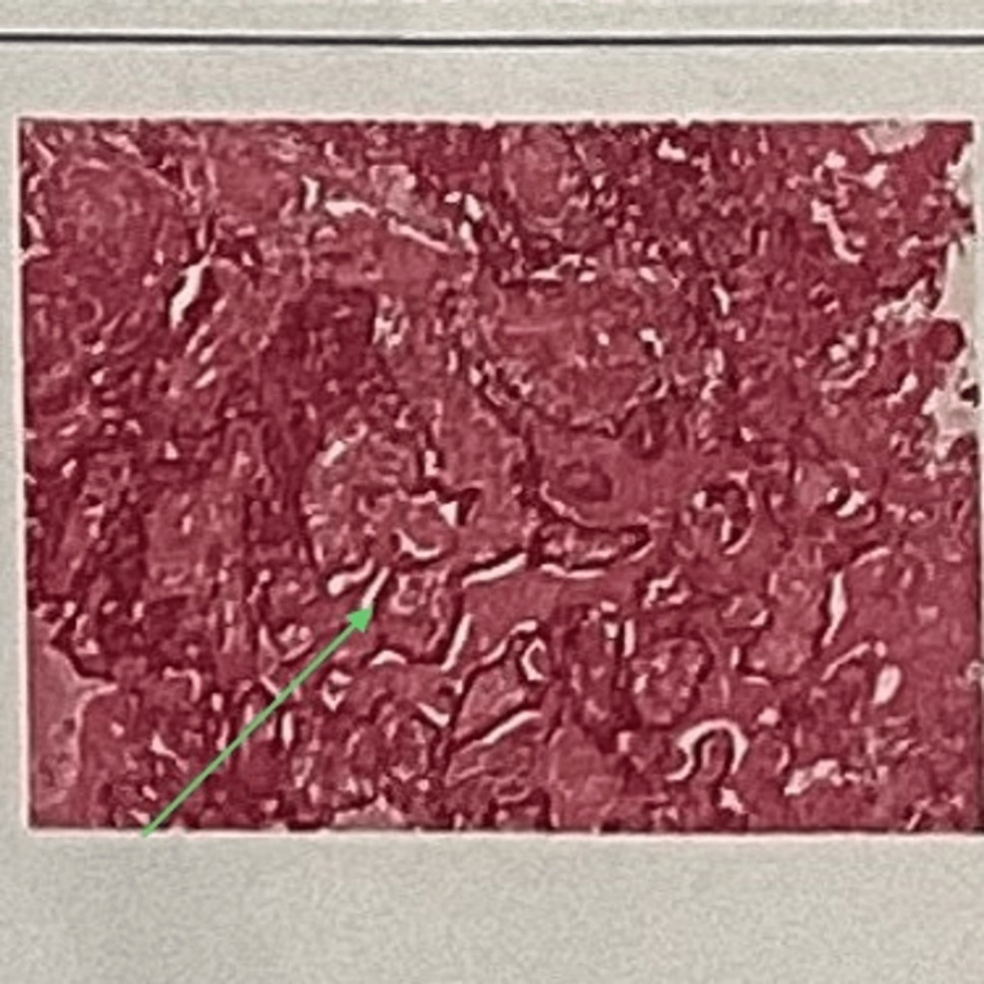
Detailed histopathological report of the incisional biopsy and the immunohistochemistry report The arrow shows epithelial tumor cells arranged in anastomosing branching cords and tubules.

Immunohistochemistry was performed for the incisional biopsy specimen (Figure 5). It was positive for pan-cytokeratin, S100, and C-Kit and focally positive for cytokeratin 7 (CK7). It was negative for smooth muscle actin (SMA), P63, and P40 for the given specimen.

**Figure 6:**
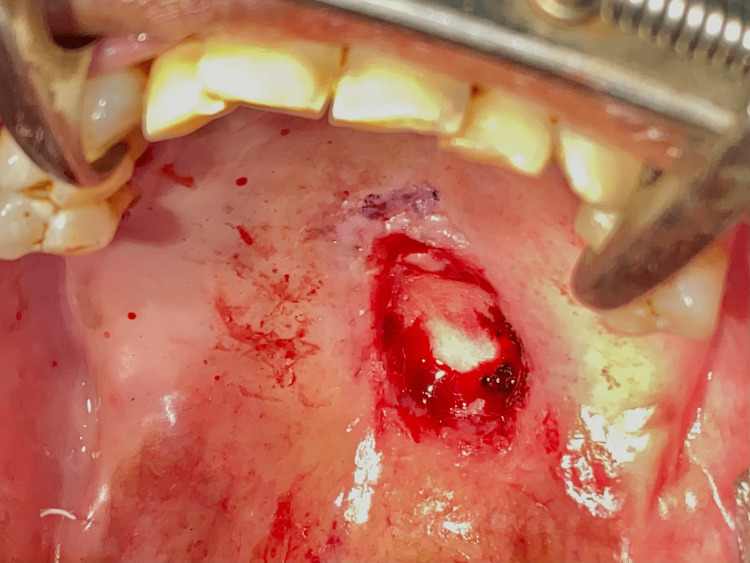
Surgical wound after excision of the lesion This image shows the postoperative surgical wound after the excision of the lesion. Note that there is no involvement of the bony palate.

**Figure 7:**
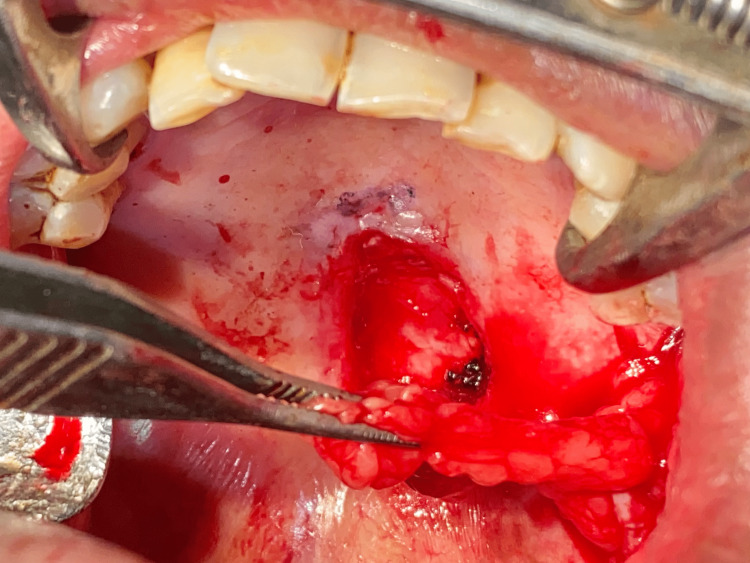
Buccal pad of fat harvest This image shows the harvest of the left buccal pad of fat to cover the surgical wound.

**Figure 8:**
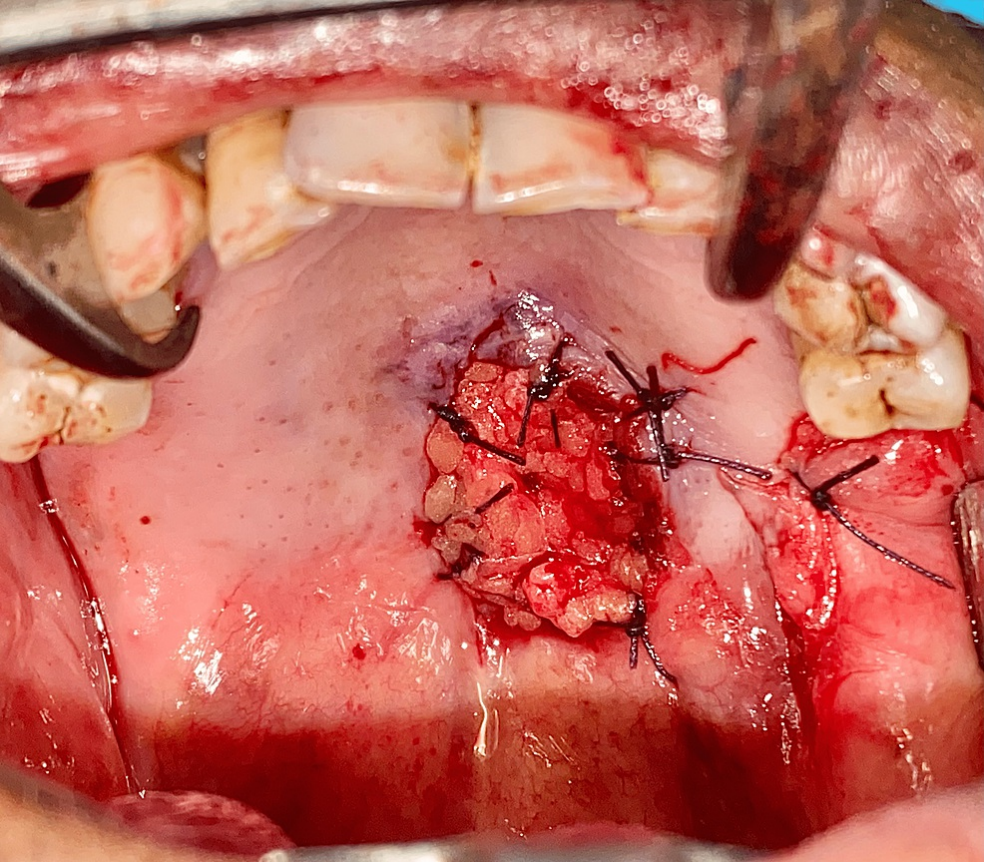
Closure of defect using the buccal pad of fat This image shows the closure of the surgical defect using the harvested buccal pad of fat.

**Figure 9:**
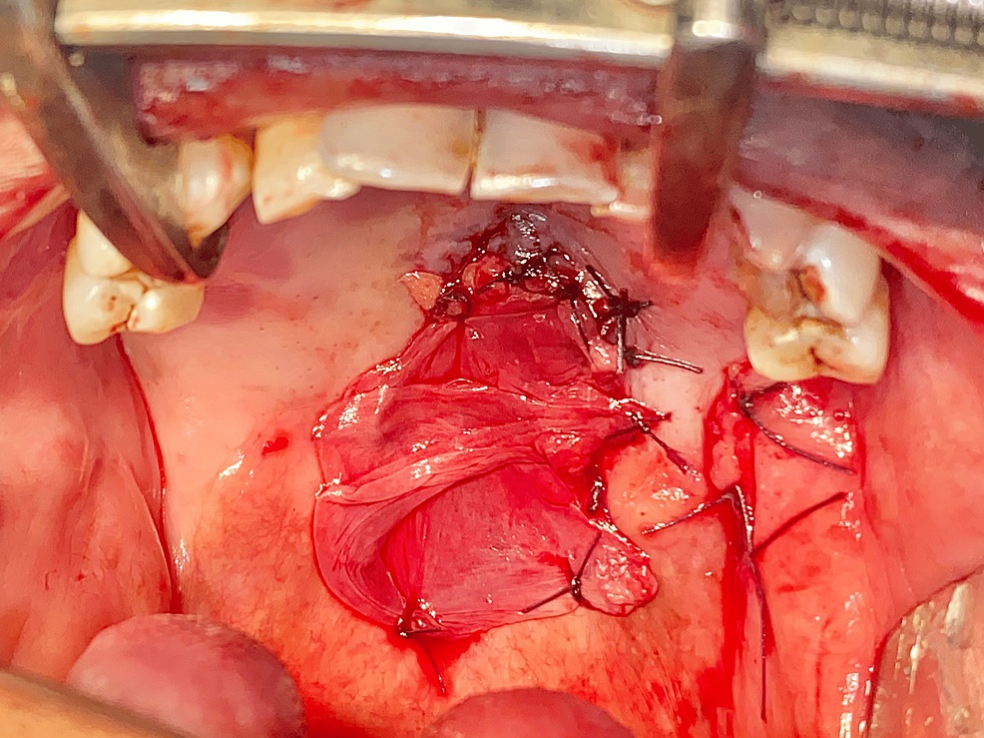
Collagen sheet placed over the buccal pad of fat

**Figure 10:**
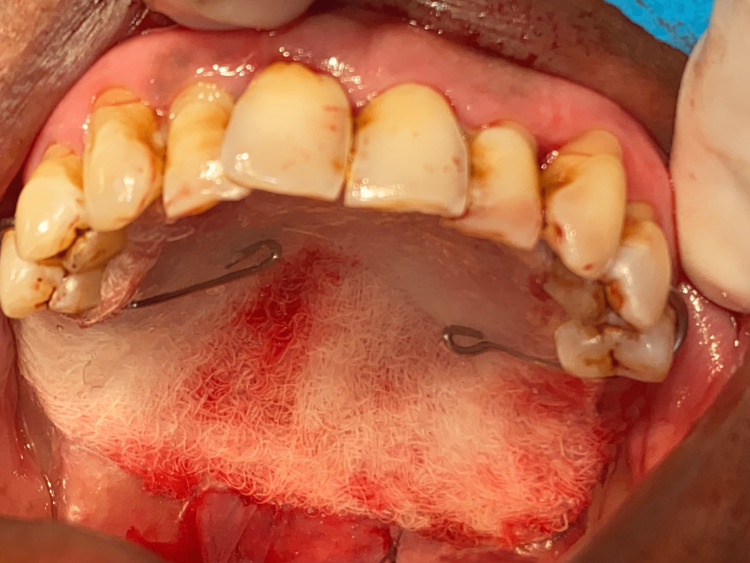
The placement of the obturator immediately after surgery

The patient was then planned for complete excision of the lesion under general anesthesia. After performing routine investigations and obtaining anesthetic fitness, the patient was planned for surgery. The patient was cooperative and understood the whole procedure. Informed consent was obtained for surgery as well as general anesthesia. Under general anesthesia, nasotracheal intubation was done. Standard scrubbing and draping were done according to protocols. Lignocaine 2% with adrenaline 1:200,000 was given as infiltration around the lesion. An incision was done using a number 15 size surgical blade. The incision was placed to remove the lesion in total. The lesion was removed and measured 2.1 x 0.9 x 0.6 cm (Figure 6). The underlying maxillary bone was found to be clear of any infiltration or ulceration. The surgical wound was closed using the harvested buccal pad of fat from the left buccal mucosa (Figure 7). The buccal fat pad was secured using 3-0 Vicryl sutures (Figure 8). A collagen sheet was placed over the fat and secured using a 3-0 Vicryl suture (Figure 9). Hemostasis was achieved. A piece of Bactigras was placed on the top and a prefabricated obturator was placed over it (Figure 10). The obturator was made using heat-activated clear acrylic resin and it is a type of immediate surgical obturator. The purpose of the obturator is to assist in swallowing, and it also serves as a matrix for the surgical dressing and provides psychological support to the patient. The patient was instructed to wear the obturator for three weeks and was advised to come for review every third day for a change of dressing. The extubation was uneventful.

**Figure 11:**
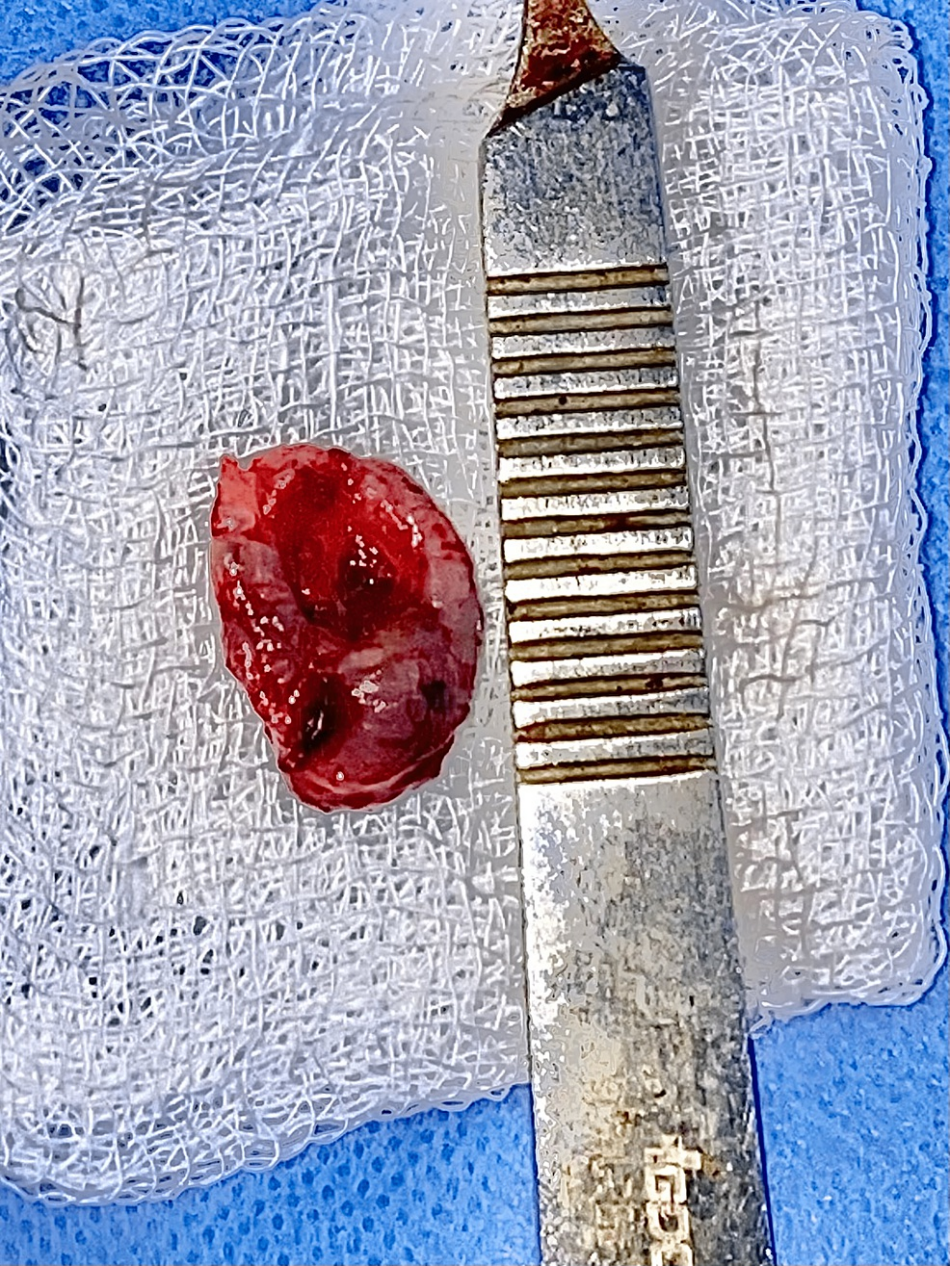
Excised lesion

The excised specimen was sent for histopathological examination and immunohistochemical analysis to the department of oral pathology (Figure 11). Surprisingly, the results turned out to be synchronous low-grade PAC with CA of the minor salivary gland of the palate. The resected base was free of the tumor with a 2 mm histological clearance.

**Figure 12:**
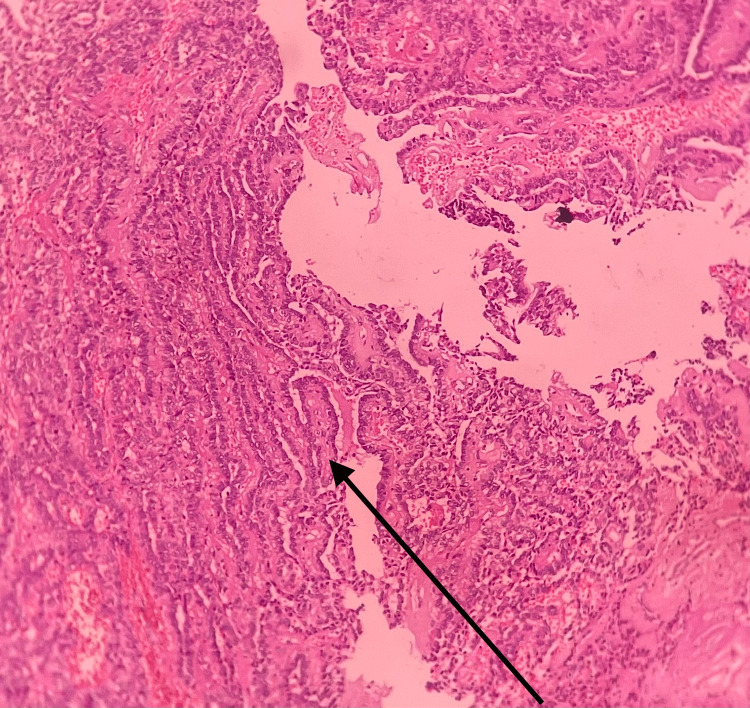
Histopathological slide of the excised specimen The image shows the cells arranged in a variegated pattern like tubular, duct-like, strands and single file arrangement with cellular uniformity.

Multiple sections showed a tumor of glandular epithelial origin, which appeared circumscribed in some areas and infiltrative in other areas. The tumor mass was composed of cells arranged in a variegated pattern like tubular, duct-like, strands and single file arrangement with cellular uniformity (Figure 12). The cells were polygonal to spindle with centrally placed round vesicular nuclei with inconspicuous nucleoli. Prominent fibromyxoid stroma was seen surrounding the tumor cells. Perineural invasion was evident. The stroma was admixed with the tumor composed of intricately anastomosing strands lined by cuboidal to columnar cells with large oval deeply basophilic nuclei. Intermittent widening and narrowing of strands were evident giving a bead-on-string appearance. The eosinophilic coagulum was noted within the lumen in a few areas. Moderate chronic inflammatory cell infiltrates along with intense vascularity, mucous acini, adipose tissue, and areas of hemorrhage were noted. Parakeratinized stratified squamous epithelium of variable thickness suggestive of the overlying epithelium was also present.

Immunohistochemistry revealed S100 was positive in tumor cells and strongly positive in CA areas. P63 showed diffuse nuclear positivity in PAC areas. P40 was negative.

Postoperative healing of the surgical site was uneventful. Complete palatal mucosa was restored and follow-up after six months revealed no signs of recurrence.

## Discussion

CA is a benign tumor of the salivary gland, which is mostly found in the minor salivary gland. It occurs on the upper lip 80% of the time, followed by the buccal mucosa and palate [4,5]. It occurs slightly more in females than males [4]. CA most commonly occurs in the fifth to seventh decade of life. It is asymptomatic and slow growing in nature. Local resection of the lesion is sufficient and shows a good prognosis.

Polymorphous low-grade adenocarcinoma is a rare, malignant salivary gland tumor, which is found almost exclusively in minor salivary glands. It is more frequent in the third to seventh decade of life, with a clear female predilection in a 2:1 ratio [6] Most of the lesions occur in the palate. Other intraoral sites can be buccal mucosa, retromolar region, upper lip, and base of the tongue. They are usually presented as a painless swelling. The overall survival of patients with PAC is generally good. Long-term follow-up is advised.

Multiple salivary gland tumors can occur as synchronous or metachronous and they can also occur unilaterally or bilaterally. The most common multiple tumors are Warthin’s tumor and pleomorphic adenoma since they have identical histological features. Multiple tumors, which are synchronous or metachronous, are uncommon. It usually occurs in major salivary glands. According to Ortega et al., their case is the first reported case of synchronous low-grade PAC and CA on the upper lip [7].

The advantages of using the buccal pad of fat for closure of the surgical wound are that the buccal pad of fat is in an anatomically favorable position, it can be harvested quite easily, has a low failure rate, and a good rate of epithelialization. The advantages of the collagen sheet are that it has chemotactic properties on wound fibroblasts, and collagen dressings encourage the deposition and organization of newly formed collagen, creating an environment that fosters healing. The disadvantage of collagen sheet dressing is that it requires a secondary dressing.

Here, we report an extremely rare case of synchronous low-grade PAC with CA of the minor salivary gland of the palate for the first time. These kinds of tumors occur very rarely. Proper examination, prompt diagnosis, and early and accurate treatment result in success and a good prognosis. The knowledge regarding the biopsy techniques and appropriate immunohistochemistry for such cases helps in proper diagnosis and treatment plans. With such a rare possibility of occurrence of these kinds of lesions, it is difficult to study the course of the disease and the effectiveness of the treatment. So, further case reports and case series need to be published to share the wisdom obtained with fellow surgeons and pathologists.

## Conclusions

To conclude, we report an extremely rare case of synchronous low-grade PAC with CA of the minor salivary gland of the palate for the first time. Even though such cases occur in rare instances, we, as oral and maxillofacial surgeons, need awareness regarding such lesions for proper diagnosis and to provide the appropriate treatment to the patients.
